# A multicenter study of short-term changes in mental health emergency services use during lockdown in Kitchener-Waterloo, Ontario during the COVID-19 pandemic

**DOI:** 10.1186/s12889-021-11807-4

**Published:** 2021-10-12

**Authors:** Christopher Dainton, Simon Donato-Woodger, Charlene H. Chu

**Affiliations:** 1 McMaster University, 1280 Main Street W., Hamilton, Ontario, 835 King St. West, Kitchener, ON Canada; 2 Grand River Hospital, 835 King St. West, Kitchener, Ontario N2G 1G3 Canada; 3 Lawrence S. Bloomberg Faculty of Nursing, University of Toronto, 155 College Street Suite 130, Toronto, ON M5T 1P8 Canada; 4 Toronto Rehabilitation Institute, University Health Network, 550 University Ave, Toronto, Ontario M5G 2A2 Canada

**Keywords:** COVID-19, Lockdown, Pandemic, Psychiatric emergency

## Abstract

**Background:**

The COVID-19 pandemic and subsequent lockdown measures have led to increasing mental health concerns in the general population. We aimed to assess the short-term impact of the pandemic lockdown on mental health emergency services use in the Kitchener-Waterloo region of Ontario, Canada.

**Methods:**

We conducted an observational study during the 6-month period between March 5 and September 5, 2020 using National Ambulatory Care Reporting System metadata from mental health visits to three regional Emergency Departments (ED); mental health and substance related police calls; and calls to a regional mental health crisis telephone line, comparing volumes during the pandemic lockdown with the same period in 2019. Quasi-Poisson regressions were used to determine significant differences between numbers of each visit or call type during the lockdown period versus the previous year. Significant changes in ED visits, mental health diagnoses, police responses, and calls to the crisis line from March 5 to September 5, 2020 were examined using changepoint analyses.

**Results:**

Involuntary admissions, substance related visits, mood related visits, situational crisis visits, and self-harm related mental health visits to the EDs were significantly reduced during the lockdown period compared to the year before. Psychosis-related and alcohol-related visits were not significantly reduced. Among police calls, suicide attempts were significantly decreased during the period of lockdown, but intoxication, assault, and domestic disputes were not significantly different. Mental health crisis telephone calls were significantly decreased during the lockdown period. There was a significant increase in weekly mental health diagnoses starting in the week of July 12 – July 18. There was a significant increase in crisis calls starting in the week of May 31 – June 6, the same week that many guidelines, such as gathering restrictions, were eased. There was a significant increase in weekly police responses starting in the week of June 14 – June 20.

**Conclusions:**

Contrary to our hypothesis, the decrease in most types of mental health ED visits, mental health and substance-related police calls, and mental health crisis calls largely mirrored the overall decline in emergency services usage during the lockdown period. This finding is unexpected in the context of increased attention to acutely deteriorating mental health during the COVID-19 pandemic.

**Supplementary Information:**

The online version contains supplementary material available at 10.1186/s12889-021-11807-4.

## Introduction

During the COVID-19 pandemic, the scope of community containment measures have been unprecedented in modern history and are colloquially referred to as “lockdowns” [1]. Such measures are variably defined, but generally expand voluntary physical distancing measures through business, school, and workplace closures, restrictions on movement and social gatherings, and imposition of legal penalties for violations. Lockdowns must often remain in place for a period of months to avoid a rebound in disease transmission among susceptible individuals when controls are lifted [2]. Quantitative evaluations of the effectiveness of such measures in attenuating pandemic peaks are rapidly emerging [3–6].

Policy considerations, however, must balance these potential benefits against direct and indirect harms. In particular, lockdowns raise ethical questions when movement is restricted in liberal democratic societies [5, 7, 8]. Community lockdowns conflict with individual rights to movement and assembly, and may further isolate the marginalized, older adults, and those living alone. This may exacerbate loneliness and depression and contribute to mental health-related morbidity and mortality [9–12]. Mental health represents an urgent health concern, and suicide mortality represents the sixteenth most common cause of death in the Waterloo Region of Ontario, Canada [13]. The direct effects of lockdown can be expected to be exacerbated by the economic stresses of business closures, increasing unemployment, and recession [12]. As a result, lockdowns make additional community support for anxiety, mental illness, suicidality, alcoholism, substance abuse, and domestic violence an ethical imperative [10, 14].

We might intuitively expect these consequences to be reflected in increased demand for and use of social and mental health services, but there has been limited formal research [10] and early reports have been conflicting. Counterintuitively, the pandemic has seen a generalized decrease in Emergency Department visits in the United States during pandemic peaks [15]. Likewise, at least three international studies suggest substantially decreased use of mental health emergency services, with one indicating a dramatically decreased number of emergency psychiatric consultations in three French centres [16], and two showing a marked decline in emergency psychiatric consultations [17] and admissions [18] during lockdown in Italy. On the other hand, European member states saw a 60% increase in emergency calls from women regarding intimate partner violence [19]. While Ontario and Durham region have seen an apparent 22% increase in domestic incidents and sexual assaults [20], women’s shelters often remain quiet [21].

The Tri-cities (Kitchener, Waterloo, Cambridge) conglomerate can be viewed as a typical semi-urban Canadian city. To better understand the short-term mental health and social impacts of public health mandated lockdowns, the primary objective of this study was to determine the short-term effects of lockdown on emergency mental health service use including: mental health related emergency department (ED) visits, mental-health related calls to police services, and calls to a central crisis help line. We hypothesized that during lockdown, there would be a year-over-year increase in use of all selected mental health services.

## Methods

All study protocols were approved by Tri-cities Research Ethics Board (THREB), as well as by the Here 24/7 Ethics Committee and Waterloo Regional Police.

### Study design

We used secondary analysis of population-level data to assess the use of multiple emergency mental health and social services during lockdown in a single region in Ontario, Canada. The observation period was over a six-month period from the region’s first confirmed case of COVID-19 on March 5, 2020 until September 5, 2020. The number of total visits were compared to the same period one year earlier in 2019.

### Population & Context

Data were captured for the Waterloo-Wellington Local Health Integration Network (LHIN), which is a region in Southern Ontario consisting of Kitchener, Waterloo, and Cambridge, with a total census population of 523,894 (as of November 26, 2020, per Statistics Canada, available at https://www12.statcan.gc.ca/census-recensement/2016/as-sa/fogs-spg/Facts-cma-eng.cfm?LANG=Eng&GK=CMA&GC=541&TOPIC=1). The region is served by three hospitals: Grand River Hospital (GRH), St. Mary’s Hospital (SMH), and Cambridge Memorial Hospital (CMH). All residents of this region with a phone would have access to police services and crisis hotlines.

In the province of Ontario, a state of emergency was declared on March 17, 2020, which directly impacted the Waterloo-Wellington LHIN. The lockdown period included school, university, and playground closures, closure of non-essential businesses, and prohibition of non-essential public gatherings of over five people followed on April 5, 2020. Graduated rollback of restrictions occurred between May 4 and June 2, 2020, with recommendations on mask wearing, social distancing, and limits on large gatherings maintained. Further details on specific restrictions can be found in Additional file 1.

### Measures of mental health related ER visits

Daily total emergency department visits were collected for GRH, SMH, and CMH between March 5 and September 5, 2020. Anonymous, retrospectively coded National Ambulatory Care Reporting System (NACRS) chart metadata for each of the three hospitals were obtained through the respective Decision Support teams mental health discharge diagnoses: substance related (excluding alcohol), alcohol intoxication, mood related (anxiety, PTSD, depression, and bipolar disorder), psychosis-related (psychosis, bizarre behaviour), situation related (situational disturbance, life crisis, concern for safety, and domestic violence), self harm related, and completion of Form 1 indicating involuntary detention for psychiatric assessment. Patients from all sites were pooled and considered as a single population. We enumerated the daily number of visits for each diagnosis over the study period. The specific ICD-10 diagnostic codes used to produce each mental health category are described in Additional file 2.

### Mental health related police service use

Waterloo Regional Police provided population-level data for the observation period and comparison period on police dispatches, as well as Neighbourhood Policing semi-monthly reports. Both sources of data were used to create the following categories: assault, domestic dispute, intoxication, and suicide attempts. We enumerated the daily numbers for each category of police call.

### Crisis-line use

Call volumes were obtained from Here 24/7, a call centre which serves as the single access point for mental health, addictions, and crisis services in the Waterloo-Wellington region. Crisis-line call volumes for Waterloo-Wellington are tracked cumulatively, and we enumerated the number of daily calls. Data on call location, presenting issues, and outgoing referral sources (i.e. emergency dispatch) were not available.

### Covid cases

Publicly available Waterloo Public Health data was used to track progression of the epidemic (as of January 19, 2021, per Region of Waterloo Public Health, available at https://www.regionofwaterloo.ca/en/health-and-wellness/positive-cases-in-waterloo-region.aspx#).

### Data analysis

Daily total Emergency Department volumes were examined between March 5 and September 5 in both 2019 and 2020. Daily new COVID-19 cases were also examined over this period in 2020. These trends were smoothed using the LOESS (locally estimated scatterplot smoothing) method [22], with a 95% confidence interval.

Descriptive analyses of the included mental health diagnostic categories were performed, and trends in the number of diagnoses in each category over time were presented visually using line graphs. Crude values are included in the Additional file 3.

ED visits, mental health diagnoses, police responses, and calls to the Here 24/7 crisis line during the first wave lockdown (March 17 to May 4, 2020) were compared to ED visits, mental health diagnoses, police responses, and crisis calls over the same period in 2019, in order to compare the lockdown period to baseline. Although data was collected from March 5 to September 5, we specifically examined the March 17 to May 4 period to study the effects of lockdown. For each category, we used a univariate quasi-Poisson regression [23] with a log link function and year as the only predictor. Quasi-Poisson models were chosen to account for the over-dispersed count data, as the residual deviance was often greater than the degrees of freedom [24]. Regression coefficients, standard error, t-values, and *p*-values are reported for each model. Statistical significance was established at *p* < 0.05. Models were created using the `glm` R function.

Significant changes in ED visits, mental health diagnoses, police responses, and calls to the crisis line from March 5 to September 5, 2020 were examined using changepoint analyses. The changepoint models found significant changes in mean and variance using the Pruned Exact Linear Time (PELT) method [25] with a range of penalties between twice the log of the number of observations and 100 times the log of the number of observations. Diagnostic plots comparing the number of changepoints and the penalty values (see Supplementary Materials) were used to find the optimal number of changepoints. We tried to find the “elbow” of these plots, minimizing both the penalty value and the number of changepoints [26]. The locations of those changepoints and the means of each segment were then calculated. Models were created using the `cpt.meanvar` function from the `changepoint` R package [27].

R (version 4.02) was used to calculate all tabulations and statistics. Code is available at https://github.com/alechay/covid19-mh

## Results

**Fig. 1:**
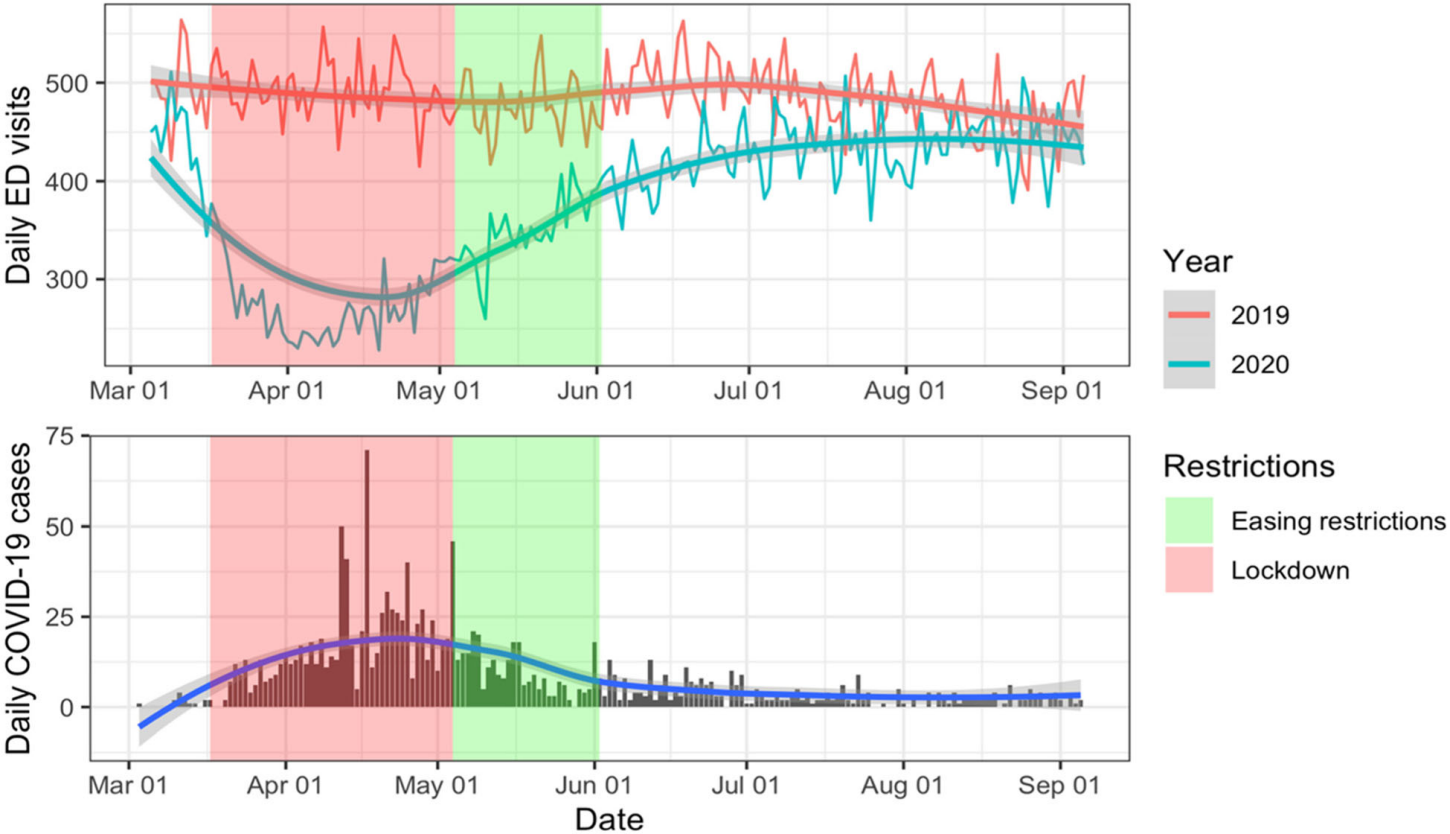
Daily total Emergency Department volumes in three hospitals in Kitchener-Waterloo and daily new COVID-19 cases in the community from March 5 to September 5, 2019–2020. Trends were smoothed using the LOESS method (see methods section), with a 95% confidence interval for predictions shaded in gray. The shaded areas indicate the periods of lockdown (red) and diminishing restrictions (green)

A total of 69,877 ED visits occurred from March 5 to September 5 in 2020, and these included 8674 visits (12.4%) related to mental health and substance use. There were 89,800 ED visits during the same period in 2019, and these included 11,045 visits (12.3%) related to mental health and substance abuse. Figure 1 shows the trend in Emergency Department total volumes between March 5 and September 5 in 2019 and 2020, along with trend in the number of daily COVID-19 cases. There was a decrease in Emergency Department volumes that mirrored the concomitant increase in COVID-19 cases and hospitalizations during the first wave of the epidemic. Emergency services and capacity to provide clinical care were not overwhelmed at any point during the pandemic first wave in the region, and there was no system-level diversion of services.

A total of 13,483 ED visits occurred during the March 17 to May 5, 2020 lockdown period, and these included about 1500 visits related to mental health and substance use. There were 24,102 ED visits during the same period in 2019, and these included about 2700 visits related to mental health and substance abuse. Quasi-Poisson regression showed that ED visits were significantly different during this period compared to the same period in 2019 (t = − 30.289, *p* < 0.001).

**Fig. 2:**
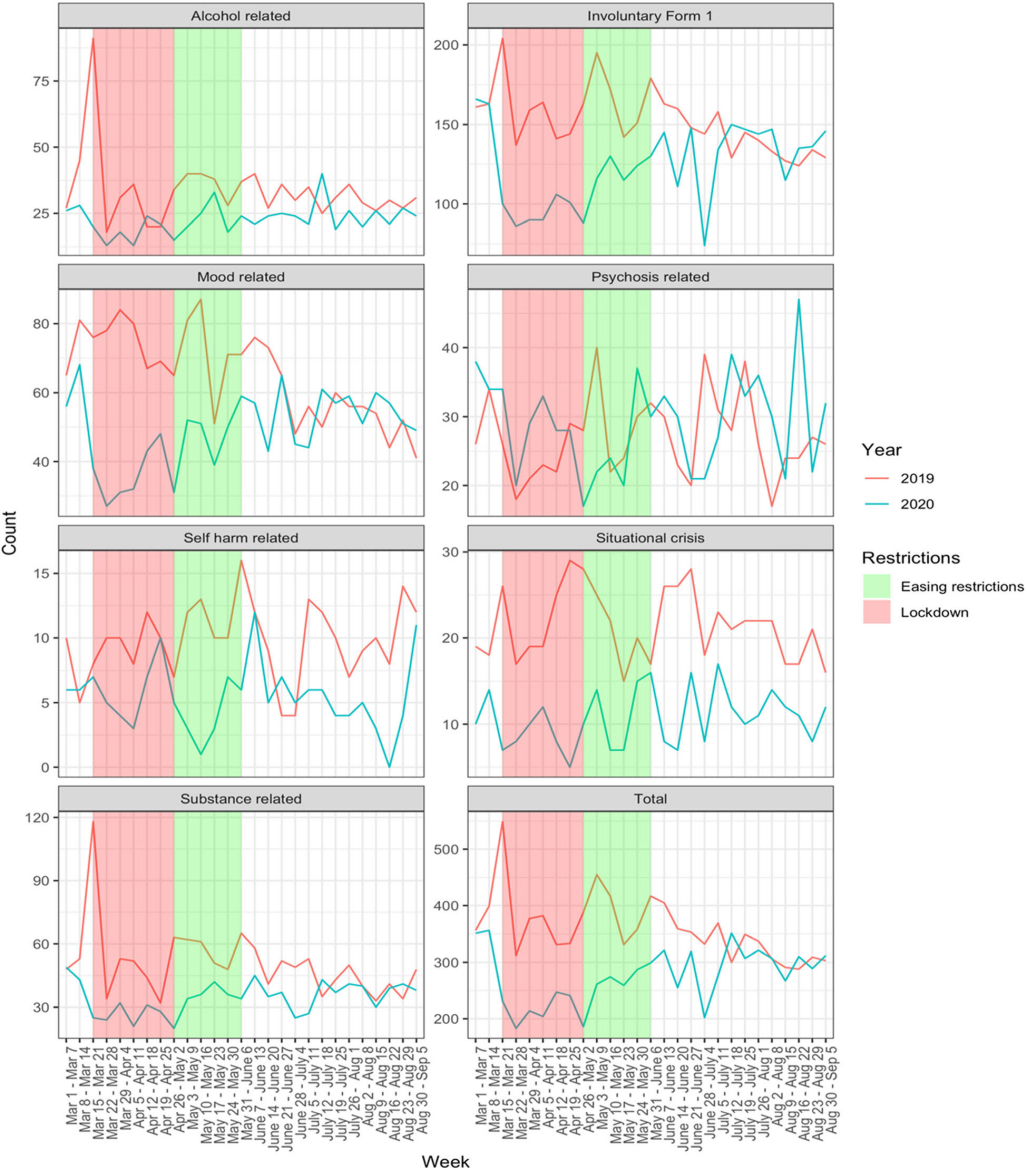
Year-over-year change between 2019 and 2020 in weekly mental health diagnoses in three Emergency Departments in the Kitchener-Waterloo region: alcohol related, involuntary Form 1, mood related, psychosis related, self-harm related, situational crisis, and substance related. Total weekly mental health diagnoses were also compared. Shaded areas indicate periods of lockdown (red) and diminishing restrictions (green)

Figure 2 shows the trend in mental health diagnostic categories in the ED over the same period and mirrors the overall trend for ED visits. The volume of each of the mental health categories were reduced during the lockdown period compared to the year before, however, only substance related, mood related, situational crisis, and self-harm related visits were significantly reduced. The number of involuntary admissions (application of Form 1) were also significantly lower during the lockdown period compared to one year earlier. Psychosis-related visits were not significantly reduced, and alcohol-related visits were reduced at a level that approached significance (Table 1).

**Table 1:** Significance of year-over-year changes in selected mental health diagnostic categories in three Emergency Departments in Kitchener-Waterloo during the lockdown period from March 17 to May 4, 2020

Type	Regression coef.	Std. Error	t value	P-val(t)
Involuntary Form 1	−0.520	0.068	−7.598	**< 0.001**
Substance related	−0.783	0.253	−3.092	**0.009**
Alcohol related	−0.701	0.340	−2.062	0.062
Mood related	−0.730	0.083	−8.845	**< 0.001**
Psychosis related	0.124	0.110	1.125	0.282
Situational crisis	−0.999	0.136	−7.333	**< 0.001**
Self-harm related	−0.461	0.158	−2.925	**0.013**
Total	−0.573	0.101	−5.687	**< 0.001**

**Fig. 3:**
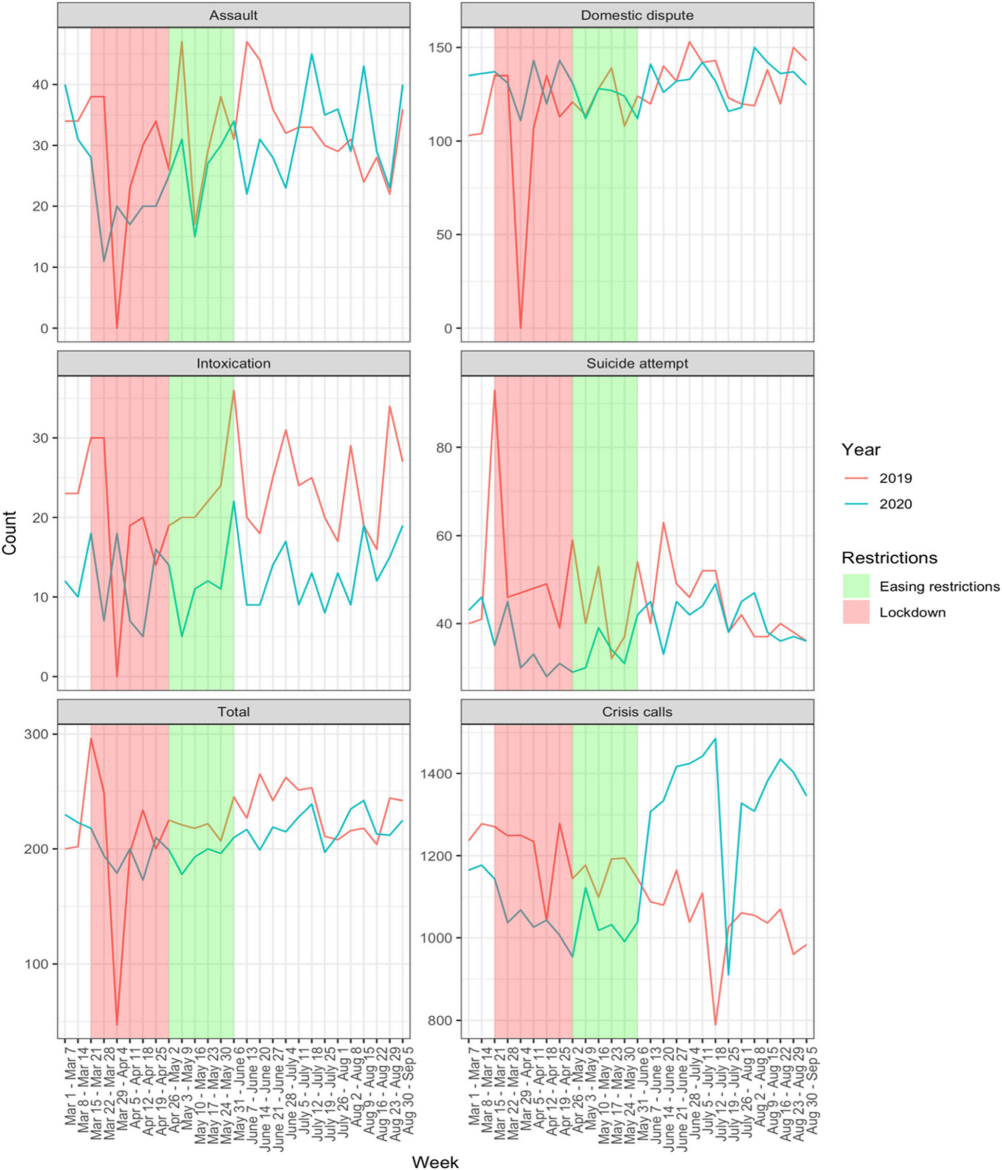
Year-over-year change in weekly Waterloo Regional Police responses between 2019 and 2020 for assault, domestic dispute, intoxication, and suicide attempts. Total police responses and calls to the crisis line were also compared. The shaded areas indicate the periods of lockdown (red) and diminishing restrictions (green)

Figure 3 shows the trend in police responses over the same period, and the trend in calls to the crisis line. Crisis calls were significantly decreased during the initial period of lockdown compared to the same period in 2019. Suicide attempts were significantly decreased during the period of lockdown compared to a year before. Intoxications, assaults, and domestic disputes were not significantly different during the period of lockdown compared to a year before (Table 2).

**Table 2:** Significance of year-over-year changes in Waterloo Regional Police response categories and calls to the crisis line during the lockdown period from March 17 to May 4, 2020

Type	Regression coef.	Std. Error	t value	P-val(t)
Suicide attempt	−0.500	0.156	−3.214	**0.007**
Intoxication	−0.440	0.281	−1.565	0.144
Assault	−0.293	0.222	−1.321	0.211
Domestic dispute	0.205	0.167	1.227	0.243
Total	−0.053	0.148	−0.360	0.725
Crisis calls	−0.151	0.035	−4.365	< 0.001

**Fig. 4:**
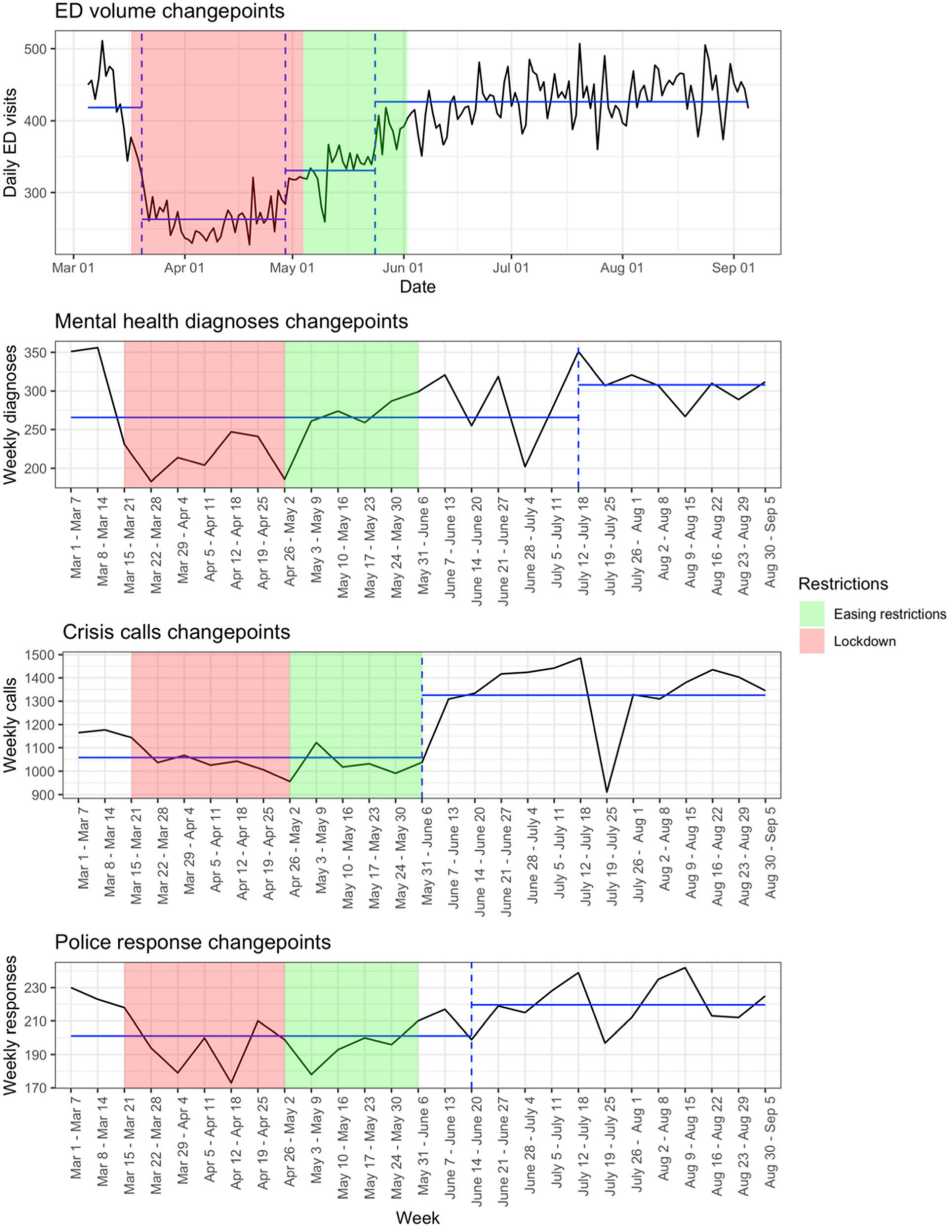
Changepoints in daily ED visits, total weekly mental health diagnoses, total weekly crisis calls, and total weekly police responses from March 5 to September 5, 2020. Changepoints indicate significant changes in mean and variance, as calculated by the PELT method. Changepoints are denoted by dashed vertical lines and segment means are denoted by horizontal lines. The shaded areas indicate the periods of lockdown (red) and diminishing restrictions (green)

Figure 4 shows the significant changes in mean and variance for daily ED visits, total weekly mental health diagnoses, total weekly crisis calls, and total weekly police responses from March 5 to September 5, 2020. There was a significant decrease in daily ED visits on March 20 and there were significant increases on April 29 and May 24, points close in time to the beginning of lockdown and the easing of restrictions. There was a significant increase in weekly mental health diagnoses starting in the week of July 12 – July 18. There was a significant increase in crisis calls starting in the week of May 31 – June 6, the same week that many guidelines, such as gathering restrictions, were eased. There was a significant increase in weekly police responses starting in the week of June 14 – June 20.

## Discussion

This study examined the association between the COVID-19 pandemic lockdown and short-term changes in emergency mental health and social services usage in a mid-sized Canadian city. Contrary to our hypothesis, we found a generalized decrease in mental health emergency visits and calls that largely mirrored the overall decrease in emergency services usage during the lockdown period. This finding is unexpected, given the current focus on acutely deteriorating mental health in various populations [11, 28], as well as fears of increased suicide rates [29].

The strengths of this study include the use of numerous independent data sources and focus on objective outcomes. It is important to note that we cannot separate the effect of pandemic itself on mental health from the effect of lockdown interventions. As an observational study, although these findings can describe trends but not infer causation, they will at least be hypothesis generating.

We can speculate that there may be several potential contributors to this decrease in demand for emergency services. First, the reduction in use of mental health services may be framed as a general reduction in hospital activities, although the concurrent reduction in police calls argues against this. There may also be a change in the threshold for involuntary admission, owing to physicians using their discretion to avoid inpatient treatment when possible. Second, the reduction in social gatherings and other opportunities for public drug and alcohol intoxication and mental health crises may have resulted in an actual reduction in the community burden of mental health morbidity. Previously public intoxications and mental health crises may have become private as a result of the closure of traditional social venues. Third, screening points limiting entry to healthcare facilities, migration of clinical care to virtual and outpatient visits, and media portrayals of overwhelmed healthcare settings may foster a perception that mental health visits are less of a priority during the pandemic [30]. In addition, there may also be a fear of contamination or infection [18] that diminishes the likelihood of the public to seek help through in-person channels, whether these be EDs or police departments.

We can also speculate on the possibility of latent and unmet demand for services by those in crisis who fail to seek treatment. In keeping with this, we note that just 5% of American students who reported increased stress and anxiety sought help through mental health counselling [28]. Similarly, reduced demand for emergency services may result from decreased reporting of mental health and social crises through traditional sources (i.e. child abuse and domestic abuse reported by teachers, family physicians, and other sources in community who may now be working virtually or not at all).

More optimistically, we cannot dismiss the role of collective resilience during the shared experience of a common disaster. Specific to the Tri-cities, we note the relatively low population density of the region, meaning that many have access to backyards, patios, and green space that may be unavailable to those living in larger and more dense urban cities. The availability of such spaces may attenuate the impact of isolation on mental health when compared to larger urban centres, promoting physical activity and improving perceived social cohesion [31].

### Limitations and future directions

This study is not without limitations. These include the inherent limitations of NACRS anonymous coded diagnostic data, which precluded detailed chart review and demographic analysis of age, gender, and ethnicity. We also lacked coroner’s data on completed suicides and deaths due to non-opioid overdoses. We cannot exclude that patients may have sought emergent help from other sources both inside and outside the community, including outpatient services, outside crisis lines and virtual resources. Similarly, it is unclear whether these results are generalizable outside the context of the Tri-cities urban region. In particular, further studies are needed to determine how the short- and longer-term effects will unfold as both the pandemic and lockdown restrictions continue in the Tri-cities region and elsewhere.

## Conclusion

This observational study provides preliminary data to quantify the early, short-term mental health and social impacts of lockdowns on demand for emergency services. These counterintuitive results may be useful in anticipating mental health emergency resource needs in future pandemic responses. The findings of decreased emergency services usage should be interpreted with caution, as they apply to the first wave of the pandemic and its initial lockdown phase, whereas many mental health emergencies may result from the incubation and accumulation of stress and isolation over a longer period. Such findings should be placed in the context of future research involving the second pandemic wave and renewed lockdown interventions.

## Additional files

**Additional file 1.**

**Additional file 2.**

**Additional file 3.**

## Data Availability

The datasets used and/or analysed during the current study are available from the corresponding author on reasonable request.
