# Supplementary material for: A multicenter study of short-term changes in mental health emergency services use during lockdown in Kitchener-Waterloo, Ontario during the COVID-19 pandemic
Source: BMC Public Health. 2021 Oct 12;21:1840. doi: 10.1186/s12889-021-11807-4 (PMC8505015; doi:10.1186/s12889-021-11807-4)
Supplement: Supplementary file 1 — Additional file 1. [file 12889_2021_11807_MOESM1_ESM.docx]

**Appendix 1**: Public health interventions in the Kitchener-Waterloo region during the first wave of pandemic lockdown in 2020.

- Three-week closure of public schools (March 12)
- University closure and prohibition of gatherings over 250 people (March 13)
- Prohibition of public gatherings over 50 people (March 16)
- Self isolation for 14 days if travel outside Canada (March 16)
- Ontario announces declaration of state of emergency and closures of public gathering places, municipal facilities and seating in bars, cafes, and restaurants (March 17)
- Closure of all non-essential businesses and cultural institutions (March 24)
- Region of Waterloo declares state of emergency (March 25)
- Prohibition of non-essential public gatherings over 5 people (April 5)
- Re-opening of garden centres, landscaping, essential construction, automatic car washes, auto dealerships by appointment (May 4)
- All construction, all retail except indoor malls (with physical distancing), vehicle dealerships, media operations, scheduled surgeries and diagnostic imaging, in-person counselling, individual sports (except high contact and team sports), general maintenance, repair, and household services (May 19)
- Resumption of elective and non-essential care (May 26)
- Increased limit on social gatherings from 5 to 10 persons, places of worship opened with 30% building capacity, personal care, shopping malls, cultural and recreational activities (June 2, excluding Greater Toronto Area)
