# Supplementary material for: A multicenter study of short-term changes in mental health emergency services use during lockdown in Kitchener-Waterloo, Ontario during the COVID-19 pandemic
Source: BMC Public Health. 2021 Oct 12;21:1840. doi: 10.1186/s12889-021-11807-4 (PMC8505015; doi:10.1186/s12889-021-11807-4)
Supplement: Supplementary file 2 — Additional file 2. [file 12889_2021_11807_MOESM2_ESM.docx]

**Appendix 2**: ICD-10 diagnostic codes used to produce each mental health category

| **Diagnostic category** | **ICD-10 codes** |
| --- | --- |
| Substance related (excluding alcohol) | F11-19, F55 |
| Alcohol related | F10 |
| Mood related (anxiety, PTSD, depression, and bipolar disorder) | F30-39, F40, F41, F42, F45, F48, F93, F98 |
| Psychosis-related (psychosis, bizarre behaviour) | F20-29, F44, R462, R451, R454, R4688 |
| Situation related (situational disturbance, life crisis, concern for safety, and domestic violence) | F43, F91, F92, F94, F95, R455, R456 |
| Self harm related | F10-19, F55, X60-69 |
