# Supplementary figures and images for: A multicenter study of short-term changes in mental health emergency services use during lockdown in Kitchener-Waterloo, Ontario during the COVID-19 pandemic
Source: BMC Public Health. 2021 Oct 12;21:1840. doi: 10.1186/s12889-021-11807-4 (PMC8505015; doi:10.1186/s12889-021-11807-4)

**Supplemental Materials**


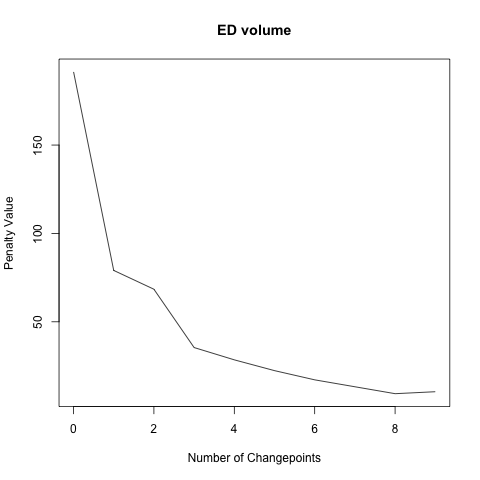

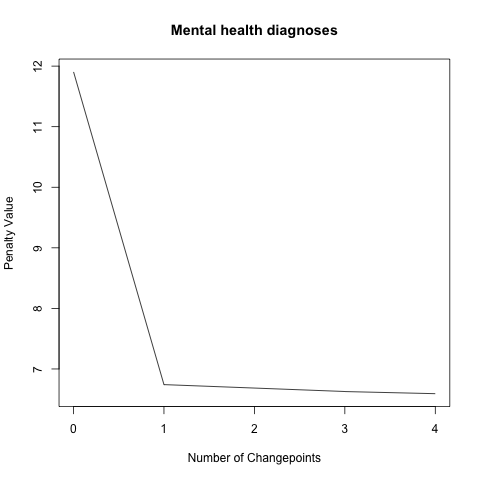


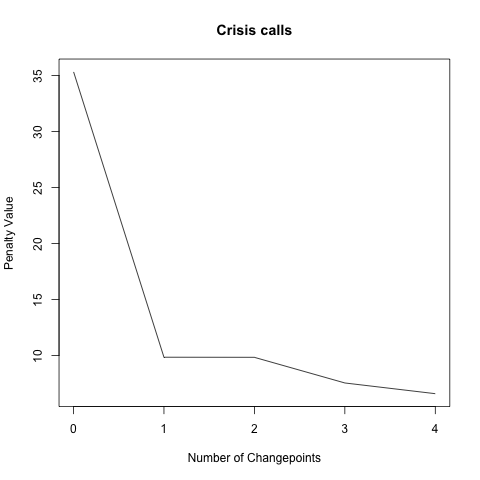

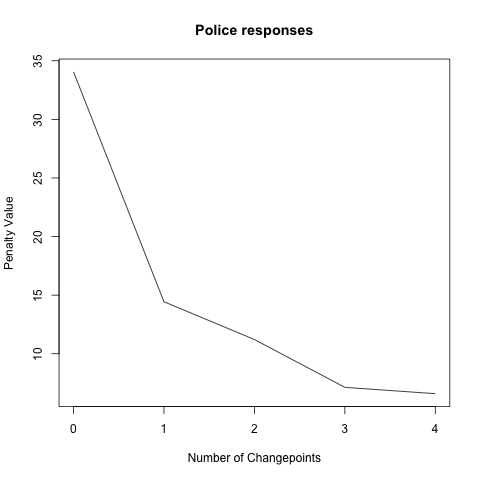

Supplement: Supplementary file 3 — Additional file 3. [file 12889_2021_11807_MOESM3_ESM.docx]
